# Can We Talk about Concordance?

**DOI:** 10.22599/bioj.370

**Published:** 2024-04-01

**Authors:** Charlotte Joy Codina

**Affiliations:** 1 University of Sheffield, UK

**Keywords:** Concordance, Patient-centred approaches, Therapeutic alliance, Prescription, Adherence, Compliance

Dear Editor,

During an orthoptic appointment, we aim to reach a treatment decision that will lead to optimal outcomes for our patients and their families. Reaching this decision requires significant clinical and communication skill, but how much involvement should patients and families have in choosing their treatment?

I frequently notice orthoptists referring to patient treatment plans using the words ‘prescribe’ and ‘comply.’ For example, a part-time total occlusion regime is prescribed for amblyopia and the individual’s compliance or adherence to that regime is reported in the patient’s notes. The terms *prescription, compliance*, and *adherence* are high-frequency words in orthoptic research and literature. The problem with the words *prescribe* and *comply* is that they imply that the patient takes orders from the health professional. These terms describe an asymmetric medical model of treatment decision reaching, where the practitioner is in control and the patient either complies or doesn’t. In wider medical literature, however, and particularly relating to medicinal treatment, these terms are superseded by the term *concordance*. Concordance describes an agreed treatment approach, reached after negotiation between a patient and practitioner that respects the beliefs and wishes of a patient in determining whether, when, and how treatment is given.

The aim of concordance is to share and agree a therapeutic decision-making process, thus empowering patients and families to choose the treatment best for them. It recognises that the health beliefs and individual circumstances of a patient and their family are important considerations. It aims to move the patient and their parent or carer from being potentially passive in their treatment to being active and informed. As such, it is associated with much better adherence to an agreed treatment plan and a wealth of literature evidences the benefit of this approach to achieving improved patient outcomes. Choosing to involve patients in their treatment options and plans, develops a collaborative therapeutic alliance, which can bring the aspirations and expectations of the orthoptist, patient, and where appropriate, their family, into alignment. It facilitates a personalised treatment approach for the patient, after dialogue and consensual agreement.

Dickinson, Wilkie, and Harris (1999), in their British Medical Journal article ‘Taking medicines: concordance is not compliance,’ advocated for the concordant treatment approach and its benefits. Yet, 25 years later, concordance is not a well-integrated term within our orthoptic community. The issue is becoming more pressing, now that the orthoptic profession has medical exemptions. Orthoptists, annotated in medical exemptions on the HCPC register, can now advise on, supply, and administer a range of exempted medicines and pharmacy only medicines. If we are to progress to independent prescribing as our profession needs, we must adopt a more progressive language, when it comes to agreeing treatment plans and medicines with our patients. If our consultation is concordant in nature, we should refer to it accordingly in our reports.

Concordance can’t be used as a synonym for compliance, because it describes something quite different. For example, ‘better concordance’ describes the mutuality of decision making between orthoptist and patient, whereas better compliance describes a closer match between the patient’s behaviour and the prescriber’s recommendations. Concordance is therefore not the same as compliance or adherence but seeks to improve both.

Concordance in medicines is a challenge, but one worth investing in, because it results in patients who are committed to seeing through the treatment plan they have chosen. Without concordance, patient treatment adherence globally, is estimated to be as low as 50% (Carter et al. 2005). Non-compliance is the most common cause of treatment failure within ophthalmology (Beery 2021). Viewing our patients and families as partners in treatment decisions is imperative to building a positive clinician-patient relationship, with effective communication key to improving patient health outcomes.

Let’s take a common orthoptic decision-making moment: a patient with anisometropic amblyopia who has finished refractive adaptation; needs further intervention to improve visual acuity. The more asymmetric, practitioner-prescribing approach would mean that the Orthoptist decides on the next treatment, for example, occlusion two hours per day for two months. A concordant approach, however, would divide the choice of treatment more symmetrically between patient, carer, and practitioner, dialoguing the treatment options available, such as atropine penalisation and part-time occlusion and reaching a mutual decision on treatment choice, with agreement and active participation from all. Similarly, regarding glasses, whilst it largely wouldn’t be appropriate for child or family to be involved in the specifics of lens prescription; concordant decisions can be reached about the need for and amount of glasses wear. This tripartite involvement will of course, not always be an equal power distribution—a teenager would be expected to have more involvement than a toddler, for example. However, concordance involves as much participation and ownership as can be advocated for.

A quick literature search of the words *orthoptics compliance* in the last five years yielded approximately 1500 articles. The search term *orthoptics adherence* yielded 1220 and *orthoptics concordance* yielded just 274. I am concerned that, even if we are embracing concordance in clinical consultations, we are not reflecting this as progressively as we might in our language. I suggest that, as a profession seeking to gain independent prescribing, we embrace a language and possibly attitudinal shift that reflects the patient-centred care, individual choice, and concordance that we seek to offer our patients.
